# Preparation and Characterization of Electrospun Collagen Based Composites for Biomedical Applications

**DOI:** 10.3390/ma13183961

**Published:** 2020-09-07

**Authors:** Mioara Drobota, Luiza Madalina Gradinaru, Stelian Vlad, Alexandra Bargan, Maria Butnaru, Marian Angheloiu, Magdalena Aflori

**Affiliations:** 1 Physics of Polymers and Polymeric Materials, “Petru Poni” Institute of Macromolecular Chemistry, Aleea Grigore Ghica Voda, 41A, 700487 Iasi, Romania; gradinaru.luiza@icmpp.ro (L.M.G.); vladus@icmpp.ro (S.V.); anistor@icmpp.ro (A.B.); mariabutnaru@yahoo.com (M.B.); 2 Department of Biomedical Sciences, “Grigore T. Popa” University of Medicine and Pharmacy, 9-13, Kogalniceanu Street, 700115 Iași, Romania; 3 Sanimed International IMPEX SRL, Sos. București Măgurele 70F, 051434 Bucharest, Romania; marian.angheloiu@sanimed.ro

**Keywords:** collagen, PET, nanofibrous mats, FTIR-ATR spectroscopy, Dynamic Vapor Sorption (DVS), cell growth

## Abstract

Electrospinning is a widely used technology for obtaining nanofibers from synthetic and natural polymers. In this study, electrospun mats from collagen (C), polyethylene terephthalate (PET) and a blend of the two (C-PET) were prepared and stabilized through a cross-linking process. The aim of this research was to prepare and characterize the nanofiber structure by Fourier-transform infrared with attenuated total reflectance spectroscopy (FTIR-ATR) in close correlation with dynamic vapor sorption (DVS). The studies indicated that C-PET nanofibrous mats shows improved mechanical properties compared to collagen samples. A correlation between morphological, structural and cytotoxic proprieties of the studied samples were emphasized and the results suggest that the prepared nanofiber mats could be a promising candidate for tissue-engineering applications, especially dermal applications.

## 1. Introduction

Electrospinning is a fiber-production technology that involves the use of a very high voltage source and does not require high-purity feed material. This method reduces the purification process of the recycled raw material prior to usage. Recent studies have presented various methods by which to obtain nanofibers, such as self-assembly, three-dimensional (3D) printing [1], template use and electrospinning. Other authors have developed methods of encapsulation with enzymes as an alternative method, with nanofibrous support showing good advantages [2,3]. Electrospinning is the preferred method among these, because it presents a cost-effective advantage and is a simple way to obtain continuous nanofibers from a large range of polymers [4]. Electrospinning is a method used under the action of an electrostatic field to produce thin fibers with nanometric or micrometric dimensions [5]. Electroplating uses polymer solutions or melts solutions [6]. The produced fibers have thinner diameters and larger surface areas in relation to volume compared with others obtained via different methods [7]. The electrospinning technique produces fibrous materials and is an alternative for obtaining nanofibers with nanometric dimensions and properties that can be evaluated and used in high performance applications [8]. These new organized polymer nanofibers have aroused great interest for biomedicine, bioengineering, pharmaceutical, and healthcare applications [9].

Collagen (C) is the most abundant fibrous protein that can be found in all organisms and is the main structural element of extracellular matrices in connective tissue [10]. This protein has a self-assembled helicoidally α triple-helix structure that contains glycine at every third position [11]. A variety of collagen structures with different functionalities can be obtained via the electrospinning technique [12]. Collagen molecules can be extracted from a wide variety of sources, but the extracting method’s sources and post-processing conditions can alter the structural and biological properties, including their biocompatibility. The new materials enriched with collagen molecules have attracted interest in a wide range of applications in the biomedical domain, and collagen fibers have been extensively studied in recent years [13]. An important challenge has been to find an adequate solvent system that can be used for the production of polymeric fibers. A most promising organic solvent that is effective for the solubilization of a wide range of polymers is hexafluoroisopropanol (HFIP), due to its vaporization temperature [14]. Concentration is therefore an important parameter in obtaining optimum conditions for the preparation of polymer fibers with nano-size diameters and without manufacturing defects [15]. Different types of collagen such as type I (from bovine skin) or type II and III (from human placenta) have been electrospun into nanofibers by taking advantage of their excellent biological properties [16]. However, the poor mechanical property of collagen scaffold limits their biomedical applications. To overcome these limitations, the properties of collagen scaffold can be improved by adding various synthetic polymers into the electrospun blend.

Polyethylene terephthalate (PET) is a thermoplastic material commonly used in different applications. This semicrystalline polyester has been widely used due to properties such as its chemical resistance, excellent tensile strength, thermal stability, low cost and ability to be electrospun [17,18,19]. For these reasons, PET nanofibers have been explored in a variety of fields including protective textiles, filtration, medicine (tissue engineering, implants, membranes and drug delivery), optical and chemical sensors, photovoltaic cells, wound dressings, defense and security and sensors [20,21,22,23].

The aim of this paper is to look at the preparation and characterization of nanofibers from synthetic and natural polymers. In order to study the phenomena that arise when these macromolecules interact with each other, we have prepared electrospinning scaffolds from collagen (C), PET and their blend (C-PET). The reason of studying the interaction between these macromolecules is that collagen can play a crucial role in biological activity because it contains specific sequences for cell attachment, while PET can support the network of the scaffold. Moreover, cytotoxicity and cell adhesion tests were performed.

## 2. Experimental

### 2.1. Materials

The solvent used for nanofibers fabrication, 1,1,1,3,3,3-hexafluoro-2-propanol (HFIP), glutaraldehyde (GA) aqueous solution (25% w/v) and absolute alcohol were purchased from Sigma-Aldrich (Belstein, Germany). Collagen type I (from calf skin) was also purchased from Sigma, St. Louis, MO, USA). Poly(ethylene terephthalate) (PET) film with a 30-μm thickness was produced by S.C. TEROM (Iasi, Romania), with 0.1% Ultrasil VN3 silica used as a plasticizer. All the reagents used for the cytotoxicity test were purchased from Sigma-Aldrich, including: Dulbecco’s Modified Eagle Medium (DMEM) culture medium with 1000 mg/mL glucose; 110 mg/L sodium pyruvate and 0.584 mg/L L-glutamine; 3-(4,5-Dimethyl-2-thiazolyl)-2,5-diphenyl-2H-tetrazolium bromide) (MTT); Hank’s phosphate buffer saline pH 7.4 (HBSS); Penicillin–Streptomycin–Neomycin (P/S/N) solution (with 5000 units penicillin, 5 mg streptomycin and 10 mg neomycin/mL), which was sterile-filtered and suitable for cell cultures; BFS (bovine fetal serum, non-USA origin), which was sterile-filtered and suitable for cell cultures; 2-propanol; Hank’s Balanced Salt Solution (HBSS) for cell cultures; and calcein AM reagent. A UV lamp was produced by Herolab GmbH (Wiesloch, Germany), which operated at 8 W power at two wavenumbers, 254/365 nm.

### 2.2. Fabrication of Electrospun Fibers

**Figure 1:**
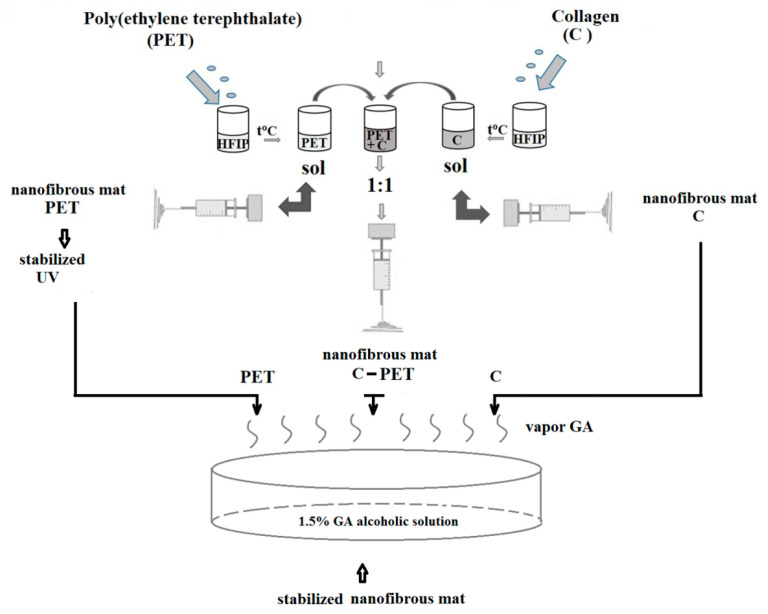
The preparation procedures for the solutions of polyethylene terephthalate (PET), collagen (C) and a combination of the two (C-PET) for electrospun fibers.

Two solutions of 10% (*w*/*w*) C and PET were prepared in hexafluoroisopropanol (HFIP) as previously [24,25]. Two solutions of 10% (*w*/*w*) C and PET were prepared in hexafluoroisopropanol (HFIP) as previously reported [24,25]. The PET solution was prepared from pieces of PET film in HFIP as a solvent, to obtain intended product. The 1-g pieces of PET film were dissolved in 10 mL HFIP, while 1 g of C was dissolved in 10 mL HFIP. The C-PET was prepared from C and PET in a 1:1 ratio (*v*/*v*). The solutions were stirred using a magnetic stirrer at room temperature for 2 h until a homogeneous solution was achieved. The polymer solutions (Figure 1) were converted into nanofibers using an electrospinning device.

The distance between the tip of the needle and the collector drum was adjusted to 10 cm to create nanofibers. The solutions were placed in a glass syringe connected to an infusion pump to control the solution feed rate at 0.002 mL/min, and the applied voltage was 12.5 kV at room temperature, with a device controlling the air and improving ventilation and acclimatization. The electrospun PET nanofibrous mat was stabilized after electrospinning, first using a UV device (lamp with 365 nm). The nanofibrous PET was stabilized with a UV-crosslinked device for 5 h, after which it was kept in vapor glutaraldehyde atmosphere for 30 min. The electrospun C and C-PET nanofibrous mats were stabilized using 25% GA aqueous solution diluted with absolute ethanol. A Petri dish was placed at the bottom of a desiccator containing 1.5% GA alcoholic solution for crosslinked nanofibrous mats, and the nanofibrous mats were kept in this atmosphere for 24 h.

In order to compare the C and C-PET fibers, a collagen film was prepared as follows: collagen type I (3 mg/mL) was dissolved in 0.02 M acetic acid and the suspension was cast on a 5 × 5 cm^2^ silicon slide and left to dry at 37 °C [26].

### 2.3. Methods and Methodologies

The Fourier-transform infrared with attenuated total reflectance spectroscopy (FTIR-ATR).spectra were collected using a Bruker LUMOS-FTIR Microscope equipped with an ATR reflection module (Attenuated Total Reflection, Ettlingen, Germany) with a diamond crystal and a single reflection at a 45° angle; OPUS 8 software was used for spectral processing. The sample surface was scanned in the 600–4000 cm^−1^ range. All spectra were collected with a good signal/noise ratio by cumulating 64 scans per each spectrum with a 2 cm^−1^ resolution.

Dynamic vapor sorption (DVS). The measurements were performed using IGAsorp equipment, including a fully automated and controlled analyzer supplied by Hiden Analytical (Warrington, UK). This device has an ultra-sensitive microbalance with a resolution of 0.1 μg for 100 mg and a maximum capacity of 200 mg. The samples were dried at 25 °C in flowing nitrogen (250 mL/min) until the weight of the sample was in equilibrium at relative humidity (RH) < 1% before performing sorption measurements. The RH was gradually increased from 0 to 90% in 10% humidity steps, with every step utilizing a pre-established equilibrium time between 3 and 5 min. Sorption equilibrium was obtained for each step. Finally, the RH was decreased and the desorption curves were registered.

The water vapor diffusion of nanofiber materials is a complex process [27], and the data were obtained from the sorption experiments at 25 °C. The diffusion coefficients were determined based on Fick’s first and second laws from kinetic sorption data. Based on Fick’s first and second laws, Crank deduced that the diffusion coefficient had a short time frame ($M_{t}$/$M_{\infty }\;$< 0.5), which is described by Equation (1) [27]:

(1) $$\frac{M_{t}}{M_{\infty }}=\frac{4}{l}\sqrt{\frac{D\cdot t}{\pi }}$$

where *M_t_* [g] is the mass of sorbed water vapor at time *t* [s], *M*_∞_ (g) is the mass sorbed at *t* = ∞, *l* (cm) is the nanofibrous mats thickness and *D* [cm^2^/s] is the Fickian diffusion coefficient. The Fickian diffusion coefficient of the water vapor in the nanofibers layer matrix was determined from the initial slope of *M_t_*/*M*_∞_ versus the *t*_1/2_ plot (extracted from kinetic sorption experiments). Over a longer period of time ($M_{t}$/$M_{\infty }\;$> 0.5), the Fickian diffusion coefficient can be deduced from Equation (2):

(2) $$\frac{M_{t}}{M_{\infty }}=1-\frac{8}{\pi^{2}}e^{\frac{-D\pi^{2}t}{l^{2}}}$$

The morphology and dimensions of electrospun nanofibrous mats were observed on a scanning electron microscope (SEM; FEI Quanta 200 scanning electron microscope; The Netherlands Company, Brno, produced in Brno, Czech Republic). The fibers’ morphologies and diameters were observed using a high-resolution scanning electron microscope. The fibers were kept intact by using a low accelerating voltage of 1 kV. The software ImageJ was applied to determine the fibers’ diameters. Measurements of nanofiber dimensions were taken from five different areas and an average was made for each dimension. Thus, the distribution of the dimensions of the fibers according to their frequencies was obtained.

An Alpha-Step D-500 Stylus Profiler KLA-Tencor (Milpitas, CA, USA) with a computerized surface and a high sensitivity was used to measure the height of the steps with an accuracy in the range of 10 angstroms–1.2 mm. The device is characterized by a recording speed of 0.10 mm/s and a loading force of 15 mg. The surface profilometry measured the average square roughness (R_a_) of the surface, reaching a filtration interval of 0.060 mm.

The tensile measurements of polymeric films were performed on an Instron apparatus (INSTRON model 3365; Universal Testing Machine, INSTRON, Norwood, MA, US) with a load cell of 500 N, using dumbbell-shaped cut samples (sample dimensions: 50 × 8.5 × 4 mm^3^). The tensile stress measurements were performed at different extension rates (from 0.5 to 10 mm/min).

Cell viability was assayed using the MTT protocol. In MTT assay, yellow water-soluble tetrazolium bromide salt (3-(4,5-dimethylthiazol-2-yl)-2,5-diphenyltetrazolium bromide) is reduced to purple insoluble formazan crystals by the enzymes of the living cells. Thus, cell viability in the cell cultures can be evaluated by measuring the amount of formazan. Viability tests were performed on the human epithelial cell line MCF-7 (purchased from ECACC cell bank and stored based on the approval of the Ethical Committee of the University of Medicine and Pharmacy Grigore T. Popa from Iasi, Romania). The monolayers of the MCF-7 cells were established in the 24-well culture plates using 2 × 10^4^ cells/well as the seeding concentration. Polymer samples with 6-mm diameters were sterilized in 70% ethanol solution, washed in HBSS and equilibrated in cell culture media. These were then added to the cell monolayers of each well of the culture plate. The triplicate for each sample was applied. The cell cultures had been incubated at 37 °C, 96% humidity and 5% CO_2_ atmosphere for 24, 48 and 72 h prior the MTT test. Briefly, for MTT test, the culture media from the culture wells were replaced with 500 μL of MTT work solution (0.25 mg/mL) in DMEM without BFS and P/S/N. The cultures with MTT were incubated for 3 h in the dark at 37 °C and 97–98% humidity. At the end of incubation, the MTT solution was replaced with the same amount of 2-propanol for the solubilization of the formazan. The absorbance of the formazan solution was quantified by a spectrophotometric measuring at λ = 570 nm using a UV/VIS Tecan plate reader (Austria). Cell viability in each experimental culture was compared to a negative control and expressed as normalized graphs. The polymers that retained at least 80% cell viability compared to those measured in the control cultures were considered non-cytotoxic (in accordance with ISO 10993-5: Cytotoxicity).

A cell adhesion test was performed using 10-mm polymeric sample discs, which were cuted using a 10-mm biopsy punch, sterilized for 40 min in 70% aqueous ethanol solution and washed twice in sterile bi-distilled water, three times in sterile phosphate buffer (HBSS-Sygma, Munich, Germany) and twice in DMEM media. After the washing procedure, each piece of material was equilibrated overnight in a 48-well culture plate, in 1 mL of DMEM supplemented with 10% BFS and 1% PSN, at 37 °C temperature and 5% CO_2_ atmosphere. A cell suspension of 2 × 10^4^ cells/well was added over each equilibrated polymer sample, achieving a final volume of 0.5 mL/well. The cell attachments on the material surfaces were recorded after 72 h of culture as microscopic fluorescent images obtained after cell staining with calcein AM vital dye. Calcein AM is a non-fluorescent cell-permeable derivative of calcein that becomes fluorescent upon hydrolysis within the cytosol of living cells. Briefly, the culture media were removed from each culture well with a polymer sample, and the polymer samples were thoroughly washed in HBSS. A volume of 200 μL of freshly made 2-μM calcein AM work solution was added over each polymer piece and incubated for 30 min at 37 °C in the dark. After the staining solution was removed, the polymer samples were washed in HBSS and analyzed with a Leica DMIL fluorescence microscope using a 455/530 nm excitation/emission UV filter. Green fluorescence highlighted living cells adhered to the material surface.

## 3. Results and Discussion

In this study, we prepared and characterized a stable nanofiber mat that was tailored using collagen (C), polyethylene terephthalate (PET) and blend of collagen and polyethylene terephthalate the two (C-PET). The study focused on a structural evaluation of the obtained nanofibrous mats using FTIR-ATR spectroscopy, as well as on the dynamic vapor sorption measurements with controlled humidity.

### Structural Characterization

Generally, Fourier-transform infrared (FTIR) data are used as molecular signatures for different materials. In this paper, the Fourier-transform infrared with attenuated total reflection (ATR) technique was used to provide information about the changes of molecular vibrations.

**Figure 2:**
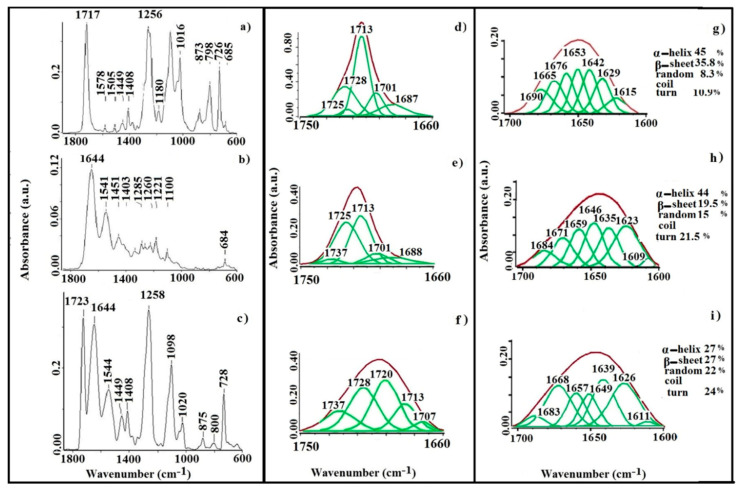
Fourier-transform infrared with attenuated total reflection (FTIR-ATR) spectra in the 1800–600 cm^−1^ range of nanofibrous mats: (**a**) PET, (**b**) C and (**c**) C-PET. Carbonyl band deconvolution in the 1750–1660 cm^−1^ range of (**d**) PET film and nanofibrous mats: (**e**) PET, (**f**) C-PET. Amide I band deconvolution in the 1700–1600 cm^−1^ range of (**g**) collagen film model and nanofibrous mats: (**h**) PET, (**i**) C-PET.

The structure of the nanofibrous mat prepared from PET, C and mixture of the two (C-PET) was evaluated, and the FTIR-ATR spectra between 1800–600 cm^−1^ are illustrated in Figure 2a–c.

Figure 2a presents the spectrum of PET nanofibers between 1800–600 cm^−1^ with the characteristic vibration bands. The structure presents signals at 1373 and 1341 cm^−1^ attributed to ɷ(CH_2_–CH_2_) from ethylene glycol that are derived from the macromolecular segment –C_6_H_4_–COO–CH_2_–CH_2_– and are characteristic of the gauche and trans conformation [28]. At 1408 cm^−1^ a complex vibration (δ(CH=CH) from an aromatic ring coupled with ν(C–C)) can be observed, which is considered to be a standard internal vibration [29,30]. The peak with the maximum at 1717 cm^−1^ is assigned to the ν(C=O) from the ester groups of the PET structure. The other characteristic vibration bands of PET are attributed at 1452 cm^−1^ to δ(CH_2_), 1180 cm^−1^ to t(CH_2_), 1016 cm^−1^ to ν(C–O), 873 cm^−1^ to the ring C–H out of the plane vibration, 798 cm^−1^ to the group (C=O+CCO) bending and at 726 cm^−1^ to the group δ(O=C–O), (δ(C=O) and δ(CH)) of the aromatic ring [31].

The FTIR-ATR spectrum from Figure 2b shows the characteristic vibration of C-fibers at 1451, 1403, 1334, 1285, 1240 and 1205 cm^−1^, which are attributed to the δ(CH_2_), δ(CH_3_), ν(C–N) and δ(N–H), respectively. There are three important vibrations of collagen molecules: at 1644 cm^−1^ assigned to ν(C=O) (amide I), 1541 cm^−1^ attributed to δ(N–H) and ν(C–N) (amide II) and at 1221 cm^−1^ corresponding to ν(C–N) and δ(N–H) (amide III). The vibrations at 1450 and 1334 cm^−1^ correspond to the stereochemistry of the proline and hydroxyproline from the collagen structure [32].

In Figure 2c the characteristic vibrations of the C-PET fibers are presented. Thus, the characteristic vibrations of both components (i.e., collagen and polyester) were detected in this spectrum. The PET structures present the characteristic absorption bands at 1723 cm^−1^ for the ν(C=O) from esteric groups, at 1408 cm^−1^ and 1449 cm^−1^ attributed to δ(CH_2_), at 1098 cm^−1^ for the νs(C–O) from the oxyethylene groups, at 1020 cm^−1^ for ν(C=C) of the aromatic ring, at 875 cm^−1^ showing the vibration of the C–H out of plane from the aromatic ring, at 800 cm^−1^ attributed to (C=O+CCO) bending vibrations and at 728 cm^−1^ for the specific group δ(O=C–O) (δ(C=O) and δ(CH) of the aromatic ring.

The collagen molecules have a strong vibration at 1644 cm^−1^ for amide I, with ν(C=O) as the principal component. The peak located at 1544 cm^−1^ is assigned to amide II, which presents a complex vibrational contribution, including the stretching ν(C=O), deformation δ(NH) and stretching ν(CN) vibrations.

Due to the collagen structure, the C-PET nanofibrous mat is enriched in –NH_2_ groups, which leads to inter- and intramolecular interactions that can be induced and influenced by their arrangement in favorable positions.

The collected data suggest that the protein reorganization is mainly a consequence of the macromolecular conformation. This behavior enables the formation of some bonds with the polyester molecules, due to the steric impediments. These interactions occur only between neighboring molecules. The vibrations at 1258 cm^−1^ correspond to amide III, and are attributed to ν(CN) and δ(NH) from the collagen molecules coupled with the vibrations attributed to ν(C(O)–O) from polyester molecules.

In order to clarify the various aspects of the structural behavior, a commercial PET film was used for the carbonyl decomposition, a specific band of the ester structure. Figure 2d–f shows the deconvolution bands of the carbonyl groups using a Lorrentian–Gaussian function in the 1660–1750 cm^−1^ region for a standard PET film (Figure 2d), PET fibers (Figure 2e) and C-PET fibers (Figure 2f).

The carbonyl vibration ν(C=O) of the ester structure of the PET film, established as a standard, is presented in (Figure 2d). This band has the maximum at 1713 cm^−1^, and its deconvolution presents many bands that are attributable to the carbonyl groups in different steric conformations in the polymer matrix. The band is a sum of all –COO– ester structures present in the macromolecular chain of the PET film. These structures determine the actual positions, which are found in different planes of symmetry in various ratios with symmetric plans of aromatic nucleus [33]. The resulting deconvolution bands are due to the distribution of the carbonyl groups in amorphous regions (1725 and 1728 cm^−1^) and in ordered regions (1687, 1701 and 1713 cm^−1^).

The electrospun PET mats present the vibration subbands of the esteric structure ν(C=O) in the same region (Figure 2e). The distribution and shape of the deconvolution bands of the carbonyl groups are different for the standard PET film compared to the electrospun PET mat. Therefore, it can be observed that the sum-band has shifted to a higher wavenumber, which is probably due to the contribution of the carbonyl groups in amorphous regions located at 1725 and 1737 cm^−1^, respectively. The integrated areas of the groups placed in amorphous regions increase while those of the ordered regions (located at 1688, 1701 and 1713 cm^−1^) decrease.

Figure 2f presents the deconvolution bands of the C-PET nanofibrous mat. It can be observed that the vibration of the carbonyl groups in the FTIR spectrum have shifted to a higher wavelength, 1723 cm^−1^, which is assigned for the new product. This intense carbonyl stretching band is probably due to the appearance of new types of carbonyl functional groups. These vibrations are attributable to the interactions between the functional groups from the polyester (acid and ketone groups) and collagen molecules. The percentage of the groups that belong to the amorphous region (1720, 1728 and 1737 cm^−1^) increases, while the percentage of groups that belong to the ordered region (1707 and 1713 cm^−1^) decreases.

A prepared collagen film was used as a model in order to determine the amide I deconvolution, because this vibration is characteristic of the structure of the protein, and is assigned to the υ(C=O) stretching vibration within the peptide bonds.

Figure 2g–i illustrates the amide I deconvolution bands in the 1600–1700 cm^−1^ region for the model collagen film (Figure 2g), PET (Figure 2h) and C-PET (Figure 2i) nanofibrous mats using a Lorrentian–Gaussian profile. These bands, which are characteristic of secondary structure, allow for identification of the components that are reported from the entire Amide I region (due to the C=O stretching vibrations of the peptide bond), and are sensitive to the conformation of the protein structures [34].

The frequencies of the component bands identified in spectrum were subsequently used as the input parameters for curve fitting of the original broad amide I band from the collagen film, which was identified as a model. The deconvolution band contained five major components attributable to the secondary structures. The percentages of each secondary structure were calculated by summing the areas for all subbands and dividing by the total area. The ranges are located at 1613–1637 cm^−1^ (*β*-sheets), 1637–1645 cm^−1^ (random coils), 1645–1662 cm^−1^ (*α*-helix), 1662–1682 cm^−1^ (turns) and 1689–1682 cm^−1^ (*β*-sheets antiparallel), respectively [35,36,37]. Figure 2g presents the vibration of the model band of the collagen film: the band at 1628 cm^−1^ is attributable to the *β*-extension intramolecular structure links, at 1644 cm^−1^ to randomly spaced spirals, at 1660 cm^−1^ to the *α*-helix, at 1675 cm^−1^ to the *β*-turns and at 1690 cm^−1^ to the antiparallel *β* sheet [38,39]. When an imino acid group is in this position prior to a glycine group, the carbonyl of the imidic group could be involved in intermolecular hydrogen bonds such as N–H (Gly) (glycine) or O=C (Pro) (proline) [40,41]. The imino acid carbonyl groups involved in the formation of hydrogen bonds are stronger, whereas amide carbonyls (which form hydrogen bonds) occur at a lower wavenumber [42].

The amide I deconvolution of the collagen-based nanofibrous mat (Figure 2h,i) indicates a percentage for the α-helix that decreased from 20.22% to 16.73% for C and was 19.81% for C-PET comparing to model carbonyl. These behaviors, it is possible, are a consequence of the new intermolecular bonds due to the secondary protein structure, as the structure of the type I collagen molecules is characterized by a triple-helix conformation. It is known from the literature data that the conformation of collagen molecules can be affected after the electrospinning process [43].

In this case, the glycine groups from the collagen molecules have a sequential position directed at the helix structure. Thus, the hydrogen bond will be carried out with carbonyl groups on the other macromolecular chain that have a perpendicular orientation positioned towards the axis of the collagen molecules.

For the C nanofibrous mat, a reduction in α-helix conformation can be observed in Figure 2h, where an increased percentage of β-sheet conformation is visible. Comparing the C-PET with the C structure of the nanofibrous mat (Figure 2i), an increase of 19.81% for *α*-helix conformation and a decrease for *β*-sheet can be observed, due to the change in the molecular order of the nanofibers. The amide I is influenced by the presence of ordered regions alternating with unordered or less ordered regions in the spatial position of carbonyl groups belonging to the collagen molecules. The *β*-sheet and unordered structure were partially converted into triple helices and *β*-sheets/aggregated strands. As a result, the content of triple-helical structures increased. As the percentage of imino acids increased, the frequency of amide I shifted to a lower wavenumber. This behavior is due to the high intensity interactions between repetitive peptide units that can be detected in folded antiparallel conformations, as well as being a result of the interactions between the carbonyls present in the tripeptide or in units adjacent to the hydrogen bonds. These observations lead to the conclusion that nanofiber preparation caused changes in the conformation of collagen molecules as compared with the model film, which is more stable due to its hydrogen bonds. The mixed nanofibrous mat had a more stable structure due to the new physical interactions between the collagen and polyester molecules.

**Figure 3:**
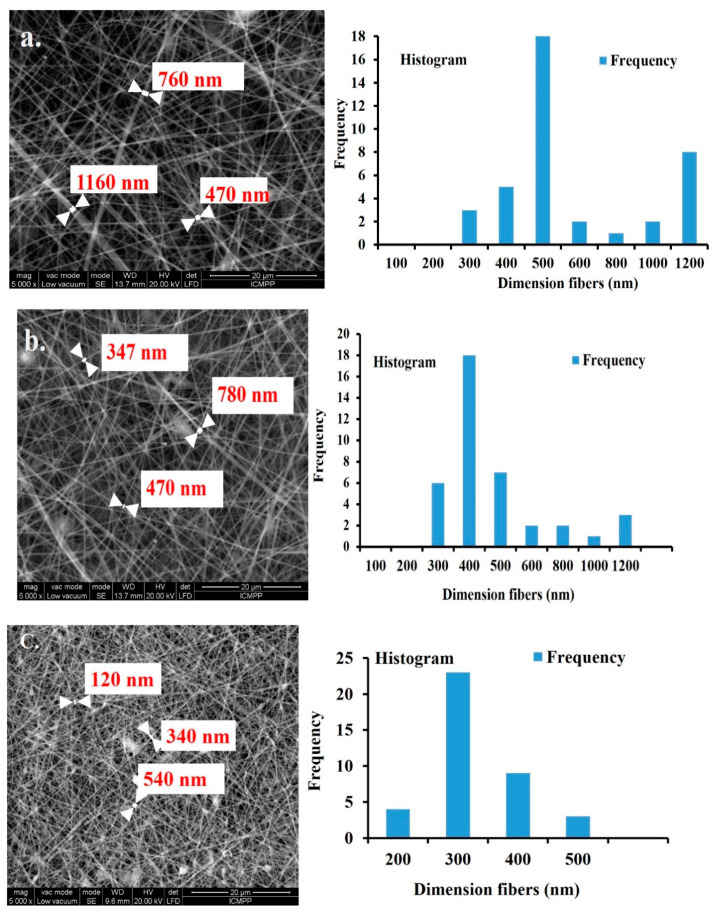
SEM image of the diameter distribution and morphologies of the nanofibrous mats: (**a**) C, (**b**) C-PET, (**c**) PET.

Scanning electron microscopy (SEM) was used to measure the fibers’ diameters. Different images for each sample were acquired and the fibers were randomly selected. The SEM images evidenced a uniform fiber texture for all samples (see Figure 3).

Fine nanofibers with uniform morphology were obtained in all nanofibrous mats.

The diameter distribution of nanofibrous C revealed higher values compared with C-PET and PET: from 470 to 1160 nm for C (Figure 3a), from 347 to 780 nm for C-PET (Figure 3b) and between 120 to 540 nm for PET (Figure 3c). The samples containing collagen presented a higher stability due to the formation of a chemical bond between the amino groups of the collagen and vapor glutaraldehyde. The active macromolecules, including peptide domains, were able to interact with collagen amine and form stable bonds. This is demonstrated by the increased fiber diameters in SEM images for samples containing collagen. This is also consistent with roughness parameters, R_a_, having values of 255 nm for C, 230 nm for C-PET and 162 nm for PET, as well as with mechanical parameters (see Supplementary Information).

The humidity parameter could be used to tune fiber diameters to the appropriate dimension for target applications, which is desirable in tissue-engineering applications [44].

The difference in fiber morphology can be attributed to the solvent evaporation process at different humidity levels. At a low humidity level, solvent evaporation is fast and a solid fiber outlayer is formed while the inner core is still a fluid. For higher humidity levels, with a sufficient processing time (to allow the solvent to evaporate completely before the dry fibers arrive at the collector electrode), cylindrical free standing fibers without the overlapping fibers can be obtained. This correlation could be explained in terms of interactions between collagen–solvent–water, collagen—solvent–PET and PET–solvent [45]. Since the solvent HFIP is miscible with water and collagen is highly hydrophilic, an increase in environmental humidity of the electrospinning process serves to allow further stretching of the liquid polymer jet in the electric field before reaching the collector. Depending on the humidity, fiber morphology can vary from ribbons to cylindrical fibers. Further increases in humidity level can result in solvent evaporation, which may induce some fibers to stick to each other on the collector [46,47].

We investigated the dynamic vapor sorption capacity and diffusion properties of water molecules into the nanofibrous materials (C, PET and C-PET) obtained by the electrospinning process. The sorption properties of the electrospun mat were compared with those of C and PET model films.

The main objective was to understand the effects of the electrospinning process on the polymer structure and the structure’s behavior under varying relative humidities at a constant temperature. It is important to know the impact of water vapors on the obtained nanofibers and the sorption/desorption mechanisms.

When hysteresis is present, the sorption and desorption rates are different. The size of the hysteresis is dependent by the nature on the components of the material. This behavior can reflect a structural and conformational reorganization/rearrangement of the components that influence the water vapor accessibility and can eventually stop the movement of the vapors. This phenomenon has been explained by many researchers such as Al Hodali, who considered a fraction with a compact structure connected using small capillary tubes [48]. In the sorption isotherm it can be seen that, according to the above mentioned studies, the capillary begins to fill as a consequence of the increase in relative humidity, while the pores are still empty. When the partial pressure of the vapors from the assigned humidity becomes higher than the vapor pressure of the liquid in the capillary, the humidity will get in the pores. In the desorption isotherm, the pore was initially full with liquid at the saturation moment. This humidity could be released only when the pressure became smaller than the vapor pressure inside of the capillary.

**Figure 4:**
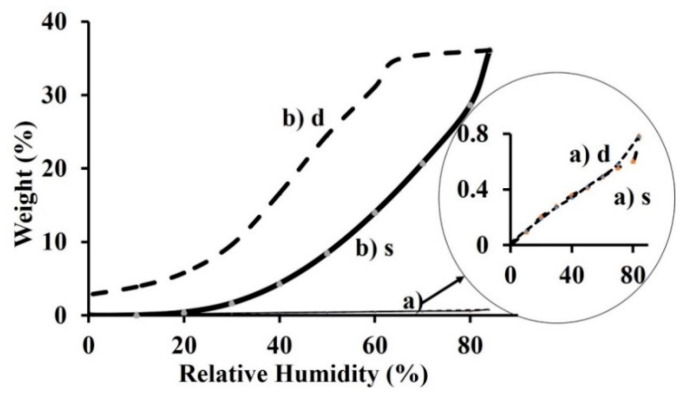
The sorption (s)/desorption (d) isotherms of (a) PET and (b) C films.

The sorption and desorption steps were closely monitored so that each recorded mass was near the equilibrium mass. It was considered that the hydration parameter measured only collagen-bound water molecules in the model film. The absorbed water content observed from DVS measurements included both bound and internal water molecules, which were sorbed inside the film (Figure 4) [49].

The divergence in response to RH changes may be reconsidered when the difference in the stage of the isotherm is observed for each measurement, and the DVS measurement includes internal free water for model collagen film. This could be anticipated under the applied experimental conditions, because the complete removal of all integral water was unlikely [50].

The proportion of free water was strongly influenced by protein macromolecules. This influence results from the proton exchange between bound and free water molecules. The water bound to protein can be divided into different types according to its state. It could be directly bonded by H-bonds to the protein both inside and outside helical fragments, and is structural water.

**Figure 5:**
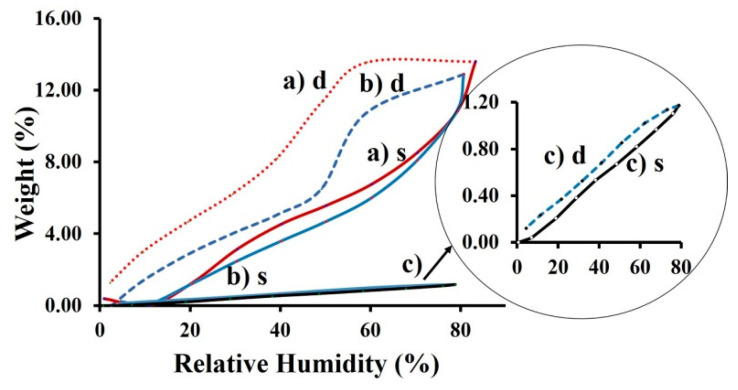
The sorption (s)/desorption (d) isotherms of the nanofibrous mats: (a) C, (b) C-PET, (c) PET.

The bound water occurs inside of the collagen triple helix and could play a stabilizing role through intramolecular hydrogen bonds that form a monomolecular layer. When the water sorbed by polar groups of collagen macromolecules from film also form strong H-bonds, it is located outside of the helical fragments contributing to the stabilization of the collagen helical structure [51]. This monolayer of water was absorbed onto hydrophilic sites from the protein, and the bonds participated substantially in the stabilization of the collagen helical structure [52]. The model isotherm is attributed to the PET model film (Figure 4a and Figure 5c).

PET is defined as a hydrophobic polyester because it sorbs less than 1% water (g/g dry), as in the curve [53]. For this reason, not many studies have been carried out to understand the sorption and diffusion of water molecules onto PET, since the majority of authors have observed a linear behavior of the water content in PET at an established relative humidity (RH) (sorption/diffusion) [54,55]. In such a case, significant changes can appear that can affect the physical properties of the polymer, such as softness and decrease of the glass transition temperature, leading to the changes in the solubility and diffusion of the penetrant [56]. These changes in isotherms are dependent on the initial microstructure and crystallinity of polymer. The shape of sorption/desorption isotherms for PET film could explain this behavior through differences in crystallinity and processing conditions compared to the C-film (Figure 4). All the fibers formed in the electrospinning process had different microstructures and crystallinity compared to the fibers obtained under standard manufacturing conditions [57]. In Figure 5, the sorption/desorption isotherm for the nanofibrous mats are illustrated.

The DVS experiments were used as a convenient way to measure the moisture uptake of samples when exposed to specified water activity levels. The obtained curves were analyzed and modeled to unravel the sorption and desorption mechanisms or modes. The optimization of the dynamic vapor sorption experiments was performed using sorption (i.e., uptake only) experimental data at a constant temperature.

The interaction with water vapors when absorbed in a polymer can generate the formation of hydrogen bonds [58,59]. These bonds can modify the diffusion of water vapors through the polymer and determine an increase in the diffusion rate. The variations in the hysteresis can be assigned to the structural swelling of the samples that contain polar groups. The structure of the C nanofibrous mat presents a higher accessibility for the water vapors compared to the C-PET mat. The shape of the sorption isotherm of the C-PET mat is similar to that of C mat, but the desorption isotherm presents a smaller delay. The C-PET is a semisynthetic material that contains a macromolecular segment from polyester that is hydrophobic, along with a second component (collagen). The collagen has hydrophilic functional groups that favor the increase of water sorption.

Water sorption can induce also changes in conformation, the hydration of polymeric matrix and transitions in amorphous phases [60,61]. In the sorption/desorption isotherms of the analyzed samples, the presence of the hydrophilic groups from the matrix of the fibers, which contain collagen molecules, increases the water vapor sorption capacity. This evolution leads to an improvement in the biocompatibility properties. According to IUPAC classification, the sorption/desorption isotherms can be associated with a type VII curve, which is characteristic of a very hydrophobic material (i.e., the PET sample). The sorption/desorption isotherms for the C-PET were similar to those of type V, which are characteristic of hydrophobic/hydrophilic materials. However, they had weak interactions and minimal water sorption at a low relative humidity, showing a moderate water vapor sorption capacity at a medium relative humidity. This can explain the phenomenon that appears in first stage of isotherm, a swelling with water vapors that appeared after completing half of the isotherm in the sorption process, where the material could store the water vapor over time. In the desorption process, the elimination of water molecules occurs due to the break of hydrogen bonds. The cleavage of these bonds is a consequence of a preliminary drying of the sample [61]. For the C nanofibrous mat, it can be observed that a high water vapor sorption appears at a relative humidity close to 90 [62,63]. Therefore, at a high relative humidity and adequate time for the C nanofibrous mat, the desorption isotherm is not closed. In this case a humidity remains in the system, and some water molecules are not desorbed.

A kinetic BET model (model for describing kinetics of physical adsorption of gas molecules on a solid surfaces) was applied to the obtained data and the calculated values are presented in Table 1. The main surface parameters of the studied nanofibrous mats were evaluated by sorption isotherms using the kinetic BET models.

This model is very often used for modeling of the sorption isotherms and is based on BET:

(3) $$W=\frac{W_{m}\cdot C\cdot \mathrm{RH}}{\left(1-\mathrm{RH}\right)\cdot \left(1-\mathrm{RH}+C\cdot \mathrm{RH}\right)}$$

where W is weight of the sorbed water, W_m_ is weight of water that forms a monolayer, C is the sorption constant and RH is relative humidity.

The BET model describes the sorption isotherms up to a relative humidity of 40%, depending on the type of sorption isotherm and the type of material. A specific surface area was calculated from the BET equation. The results from Table 2 estimate the values of the diffusion coefficients calculated from the kinetic model in the following order: C > C-PET > PET.

**Figure 6:**
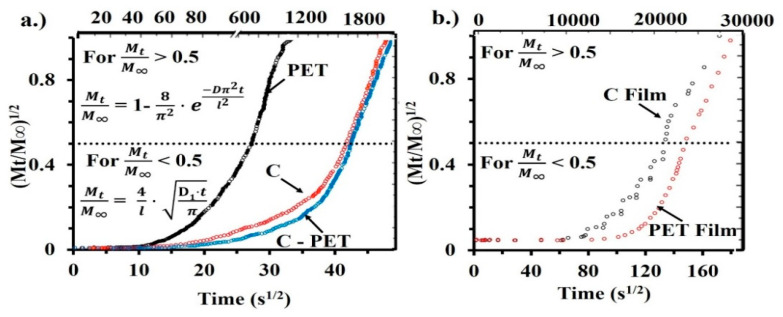
Graphical representation of ($M_{t}$/$M_{\infty })1/$^1/2^ mass changing vs. time^1/2^ of (**a**) PET, C and C-PET nanofibrous mats and (**b**) PET and C model films.

These values increased slowly due to the structures of the nanofibrous mats (Figure 6a).

In the first stage of the water vapor diffusion process, the diffusion coefficients were smaller, even at an order of magnitude comparable to the diffusion coefficients of the longest diffusion times. In Table 2 we can see a remarkable influence on the nanofibrous mat by the collagen molecules—which are also combined with the PET polyester (C-PET) and compared to an isolated PET sample—at a short diffusion time (the PET presents 1.15 × 10^−6^ and the C-PET is 0.292 × 10^−6^); at longer times, however, the contribution is minor (the values are close for all nanofibers samples: 6.41 × 10^−6^ to the 4.86 × 10^−6^).

The diffusion process was slower for the model film samples than for the nanofibrous mats. Thus, the diffusion coefficient of the PET film in the first stage is D_1_ = 0.0372 × 10^−6^ and D_2_ = 0.222 × 10^−6^, while for the PET nanofibrous mat it is D_1_ = 1.15 × 10^−6^ and D_2_ = 6.41 × 10^−6^, respectively. A similar behavior was observed in the case of the C nanofibrous mat and film. The mechanical properties of C-PET nanofibrous mat were improved too, in comparison with the C-nanofibrous mat (see Supplementary Information).

**Figure 7:**
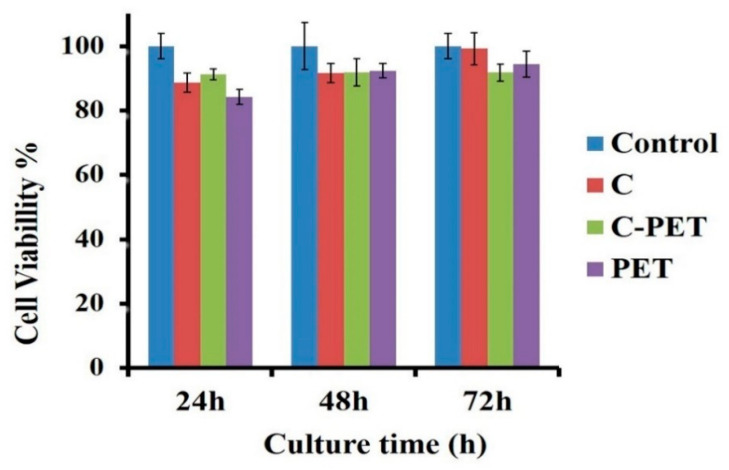
Proliferation profiles of cells grown on electrospun samples up to three days.

The MTT test for cytotoxicity indicated over 80% of the cell viability for the fibrous mats. This test indicates that the fibrous mats can support the proliferation of cells and indicate a non-toxic composition for all materials (Figure 7).

Keeping the cell viability over 80% for the three-day cultured period, all evaluated samples revealed non-cytotoxic properties, which is a good trend for the collagenous materials designed for cellular attachment and the promotion of cell spreading. According to Figure 7, cell viability increased from 89% at 24 h to 100% at 72 h in the presence of the C nanofibrous mat. For the PET nanofibrous mat, cell viability was maintained at around 89%, while the C-PET nanofibrous mat showed an increase from 87% at 24 h to 94% at 72 h. Based on these preliminary experiments, we can conclude that these nanofibrous mats could be suitable for a variety of applications in tissue engineering as scaffolds to improve cell–scaffold interaction or patches [64].

With micrometer diameters, the architecture of the fibers from the obtained networks induced cell adhesion and presented complex mechanisms of cell growth. The direction of cell growth indicates cell migration along the axis of collagen alignment [65,66], where the cells meets fibers in the nanometer dimension. Londono et al. reported that individual cells are promoted to grow by grooves [67], while Teixeira et al. presented a mechanism guidance of epithelial cells using micro- and nanostructured grooves [68].

**Figure 8:**
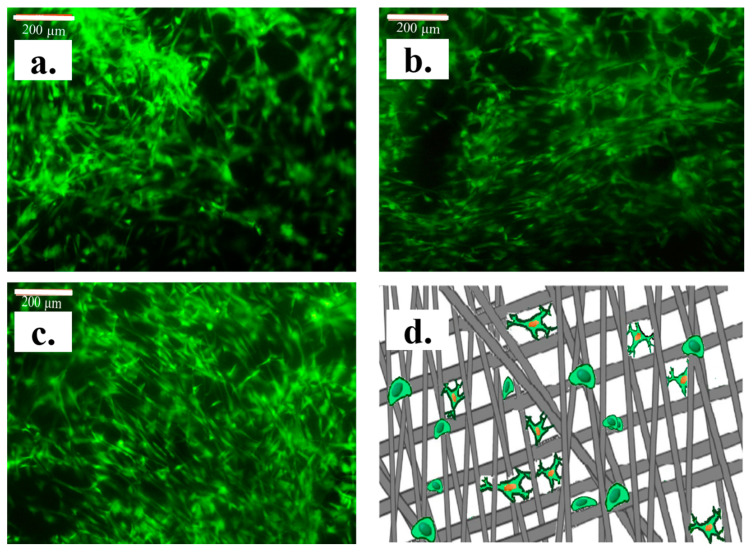
Cells cultured on nanofibrous mats of (**a**) C, (**b**) C-PET and (**c**) PET. (**d**) Schematic mechanism of proliferated cell growth.

The images of cell growth on fibrous mats shown in Figure 8a–d reveal the schematic mechanism of proliferated cell growth [69].

Cell adhesion to fibrous networks is influenced by the presence of collagen in the structure, which bridge large distances between fibers and fiber diameters. All obtained electrospun scaffolds showed a very good biocompatibility and facilitated cell attachment and proliferation. For C-PET, the fiber size and interconnection of the two polymers led to a stabilized structure, and cell growth was observed on a much denser structure than C, which had fiber dimensions up to the value of 780 nm. For the C-PET, the fiber size and interconnection of the two polymers led to a stabilized structure, and cell growth was observed on a denser structure with fiber dimensions up to a value of 780 nm; as in the case of collagen fibers, the cells grew mainly along the fiber. For the fibrous PET mat had fibers with values of diameters up to 540 nm, as well as a dense structure promoted by the cellular morphology and cell proliferation on the surface of the fiber as far as the distance between the fibers allowed.

The morphology of fibrous mats promotes cell proliferation and development in microscale and nanoscale architectures for tissue-engineering applications. As shown in Figure 8d, cells exposing additional binding sites on fibrous mats provide a difference, as cells that grew on fibers with micron dimensions presented a stretched surface and seemed to grow as they did when they were on a flat surface, while the cells grown on nanometer-sized fibers induced a mechanism of cell adhesion over a large area, with several trapped binding points of cell membrane receptors. Another aspect is the free volume given by the obtained nanofiber structure.

Nanofibrillary materials can be used as patches and have good potential in the field of cancer therapies as a drug delivery system. An important role is played by the retention of the drug when it diffuses into the cells, by using the high permeability of the skin. With an adequate drug, the novel material could ensure a good response in therapeutic treatment in stimuli-responsive controlled delivery drug applications for breast cancer therapy [70].

## 4. Conclusions

It is known that native type I collagen containing amino acid sequences is widely used as a biomaterial for tissue-engineering applications. These sequences present functional groups that lead to inter- and intramolecular interactions. By using a suitable solvent, collagen can generate fibers in electric fields with a large surface-to-volume ratios. However, low mechanical properties are a disadvantage of collagen as a biomaterial. For this reason, we have developed a semisynthetic material (obtained by a novel configuration of electrospinning) that combines the properties of collagen with those of polyethylene terephthalate. The assembly of the involved macromolecules and the factors responsible for the stabilization of the obtained structure were studied by FTIR-ATR, and the values of the diffusion coefficients of the nanofibrous mats from the dynamic vapor sorption–desorption study were demonstrated in the following order: C > C-PET > PET. The swelling measurements of the C-PET nanofibrous mat performed in the same study indicate that the functional groups interact also with water molecules. The preliminary cytotoxicity study revealed that C-PET nanofibrous mats are not cytotoxic, and can therefore be successfully used in tissue-engineering applications.

In conclusion, by using novel and improved processing techniques, materials with advanced structures can be prepared. These allow the production of fibrous mats with targeted properties to promote adequate cellular activity for biomedical applications.

## Figures and Tables

**Table 1:** Parameters evaluated using the kinetic model.

Sample	Weight (%)	BET	
		Area (m^2^/g)	Monolayer (g/g)
**PET**	1.4341	22.91	0.01183
**C**	20.6061	221.08	0.06295
**C-PET**	17.0599	143.852	0.04097

BET for the studied samples.

**Table 2:** Diffusion coefficients of all nanofibers and model film samples.

Sample	M_t/_M_∞_ < 0.5	M_t/_M_∞_ > 0.5
	D_1_ (cm^2^/s) × 10^−6^	D_2_ (cm^2^/s) × 10^−6^
PET fibers	1.15	6.41
C fibers	0.409	4.87
C-PET fibers	0.292	4.86
PET Film	0.0372	0.22
C Film	0.0298	0.38

## Supplementary Materials

The following are available online at https://www.mdpi.com/1996-1944/13/18/3961/s1, Figure S1: Roughness measurements for the studied samples (a) C, (b) C-PET, (c) PET, Figure S2: Mechanical properties, Table S1: Mechanical properties of the fibrous mats.
