# Supplementary material for: Preparation and Characterization of Electrospun Collagen Based Composites for Biomedical Applications
Source: Materials (Basel). 2020 Sep 7;13(18):3961. doi: 10.3390/ma13183961 (PMC7559754; doi:10.3390/ma13183961)
Supplement: Supplementary file 1 [file materials-13-03961-s001.pdf]

materials

MDPI

Supplementary Information

Preparation and Characterization of Electrospun Collagen Based Composites for Biomedical Applications

Roughness Measurements

The roughness parameter of the surfaces of all samples was measured with a stylus profiler as described in the main paper and the results are presented in Figure S1.

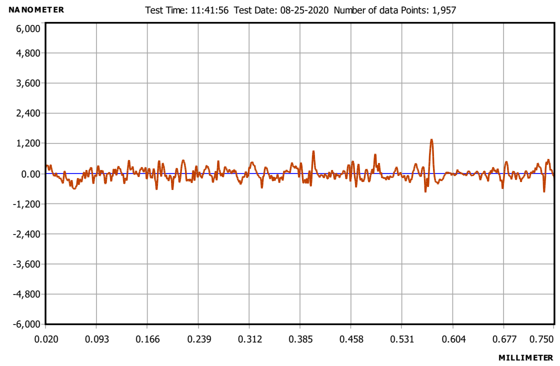

(a)

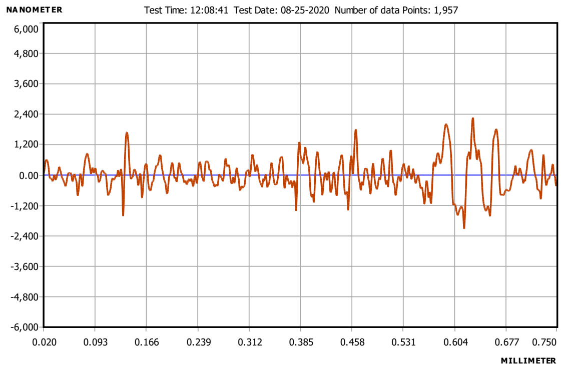

(b)

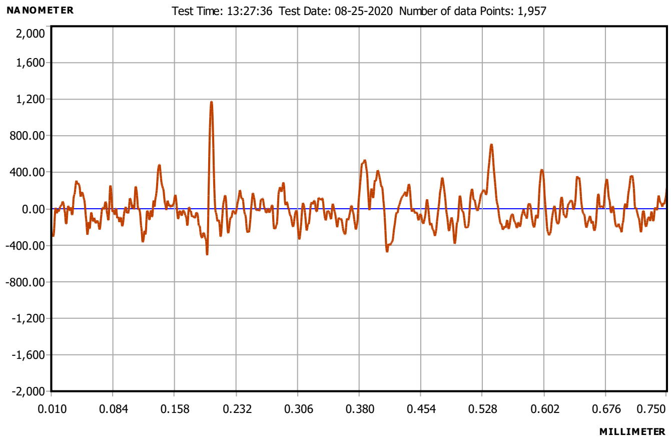

(c)

Figure S1. Roughness measurements for the studied samples (**a**) C, (**b**) C-PET, (**c**) PET.

The roughness parameter R_a varies from 255 nm for C (Figure S1a), to 230 nm for C-PET 9 (Figure S1b) and to 162 nm for PET (Figure S1c).

Mechanical Properties

The mechanical properties of nanofiber mats were measured by uniaxial tensile tests and the results are presented in Figure S2 and Table S1.

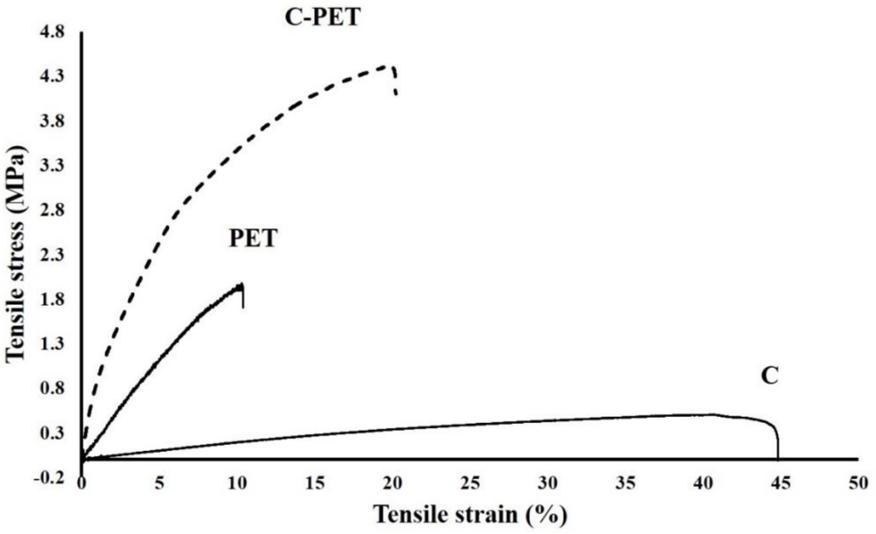

Figure S2. Mechanical properties.

Table S1. Mechanical properties of the fibrous mats.

Sample	Young's Modulus (MPa) × 10^{-3}	Elongation at Break (%)	Breaking Resistance (MPa)	Toughness at Break (MJ/m³)
C-PET	46.3	20.18	4.36	63.87
C	54.2	10.32	1.91	7.93
PET	1	44.83	0.447	18.05

The mechanical properties of the nanofiber samples were influenced by the electrospining process. From the stress–strain graph, the tensile toughness can be calculated. This parameter represents the amount of energy per volume. The tensile strength indicates how much force the material can support, and tensile toughness indicates how much energy a material can absorb before breaking.

The mechanical properties of the fibrous mats are summarized in Table S1: PET has a medium value of deformation energy 18.03 MJ/m^3, while the combination of C with PET has the highest amount 63.8 MJ/m^3. It can be seen that C-PET combines the properties of both materials and gains quality by a high breaking resistance 4.36 MPa, compared with PET (0.446 MPa) or C (1.91 MPa).
